# Supplementary material for: Performance of mid-upper arm circumference as a screening tool for identifying adolescents with overweight and obesity
Source: PLoS One. 2020 Jun 23;15(6):e0235063. doi: 10.1371/journal.pone.0235063 (PMC7310830; doi:10.1371/journal.pone.0235063)
Supplement: S1 Table — (DOCX) [file pone.0235063.s003.docx]

Table 1. Ability of MUAC to classify overweight and obesity among male adolescents, Addis Ababa,2019 (n=456)

| **Age in Years** | **AUC** | **SE** | **95% CI** |
| --- | --- | --- | --- |
| 15 | 0.99 | 0.1 | (0.97, 1.00) |
| 16 | 0.97 | 0.01 | (0.94, 1.00) |
| 17 | 0.95 | 0.04 | (0.88, 1.00) |
| 18 | 0.94 | 0.02 | (0.90, 0.98) |
| 19 | 0.94 | 0.06 | (0.83, 1.00) |

AUC, Area Under Curve; SE, Standard error; CI, Confidence interval
