# Supplementary material for: Performance of mid-upper arm circumference as a screening tool for identifying adolescents with overweight and obesity
Source: PLoS One. 2020 Jun 23;15(6):e0235063. doi: 10.1371/journal.pone.0235063 (PMC7310830; doi:10.1371/journal.pone.0235063)
Supplement: S2 Table — (DOCX) [file pone.0235063.s004.docx]

Table 2. Ability of MUAC to classify overweight and obesity among female adolescents, Addis Ababa,2019 (n=395)

| **Age in Years** | **AUC^1^** | **SE^2^** | **95% CI^3^** |
| --- | --- | --- | --- |
| 15 | 1.00 | 0.00 | (0.99, 1.00) |
| 16 | 0.93 | 0.02 | (0.88, 0.99) |
| 17 | 0.94 | 0.03 | (0.89, 0.99) |
| 18 | 0.96 | 0.02 | (0.92, 1.00) |
| 19 | 1.00 | 0.00 | (1.00, 1.00) |

AUC, Area Under Curve; SE, Standard error; CI, Confidence interval
