# Supplementary material for: Performance of mid-upper arm circumference as a screening tool for identifying adolescents with overweight and obesity
Source: PLoS One. 2020 Jun 23;15(6):e0235063. doi: 10.1371/journal.pone.0235063 (PMC7310830; doi:10.1371/journal.pone.0235063)
Supplement: S3 Table — (DOCX) [file pone.0235063.s005.docx]

Table 3. Sensitivity, speciﬁcity, positive predictive value, negative predictive value, positive likelihood ratio, negative likelihood ratio, Youden index and optimal cut-off values of mid-upper-arm circumference in predicting overweight (including obesity) in adolescent males (n=456)

| **Age** | **Sensitivity (%)**  (95% CI) | **Specificity (%)**  (95% CI) | **PPV**  **(%)**  (95% CI) | **NPV**  **(%)**  (95% CI) | **LR+**  (95% CI) | **LR−**  (95% CI) | **Youden index** | **Cut off point**  **(cm)** |
| --- | --- | --- | --- | --- | --- | --- | --- | --- |
| 15 | 100  (69.2-100) | 97.9  (96.6-98.7) | 89  (83.6-92.7) | 100  (.-.) | 46.7  (29.6-73.8) | 0  (0-0) | 0.97 | 27.7 |
| 16 | 90  (68-98.8) | 96  (94.5-97.3) | 79.6  (73.1-84.9) | 98.2  (93.7-99.5) | 22.7  (15.7-32.6) | 0.10  (0.03-0.39) | 0.84 | 27.6 |
| 17 | 87.5  (47.3-99.7) | 95.4  (93.7-96.7) | 76.5  (68.5-83) | 97.8  (87.6-99.6) | 18.9  (12.6-28.3) | 0.13  (0.21-0.82) | 0.84 | 27.5 |
| 18 | 100  (66.4-100) | 95.6  (94-96.9) | 79.7  (74.1-84.3) | 100  (.-.) | 22.8  (16.6-31.2) | 0  (.-.) | 0.88 | 28.5 |
| 19 | 100  (39.8-100) | 99.5  (98.8-99.9) | 97.3  (93.2-99) | 100  (.-.) | 212  (79-163) | 0  (.-.) | 0.87 | 27.8 |

CI, confidence interval; LR+, positive likelihood ratio; LR-, negative likelihood ratio, NPV, negative predictive value; PPV, positive predictive value
