# Supplementary material for: Performance of mid-upper arm circumference as a screening tool for identifying adolescents with overweight and obesity
Source: PLoS One. 2020 Jun 23;15(6):e0235063. doi: 10.1371/journal.pone.0235063 (PMC7310830; doi:10.1371/journal.pone.0235063)
Supplement: S4 Table — (DOCX) [file pone.0235063.s006.docx]

Table 4. Sensitivity, speciﬁcity, positive predictive value, negative predictive value, positive likelihood ratio, negative likelihood ratio, Youden index and optimal cut-off values of mid-upper-arm circumference in predicting overweight (including obesity) in adolescent females (n=395)

| **Age** | **Sensitivity (%)**  (95% CI) | **Specificity (%)**  (95% CI) | **PPV**  **(%)**  (95% CI) | **NPV**  **(%)**  (95% CI) | **LR+**  (95% CI) | **LR−**  (95% CI) | **Youden index** | **Cut off point**  **(cm)** |
| --- | --- | --- | --- | --- | --- | --- | --- | --- |
| 15 | 100  (79.4-100) | 98.2  (97.1-99) | 90.6  (85.3-94.1) | 100  (.-.) | 90.6  (85.3-94.1) | 0  (.-.) | 0.99 | 27.5 |
| 16 | 100  (81.5-100) | 93.9  (92-95.4) | 73.8  (68.3-78.6) | 100  (.-.) | 16.3  (12.5-21.3) | 0.0  (0.0-0.0) | 0.75 | 26.6 |
| 17 | 93.3  (68.1-99.8) | 93.9  (92.1-95.4) | 72.5  (65.2-78) | 98.8  (82.5-99.8) | 15.3  (11.4-20.6) | 0.07  (0.01-0.47) | 0.73 | 26.4 |
| 18 | 95.2  (76.2-99.9) | 95.8  (94.2-97) | 79.6  (73.5-84.5) | 99.2  (94.5-99.9) | 22.6  (16.1-31.7) | 0.04  (0.01-0.33) | 0.84 | 27.9 |
| 19 | 100  (15.8-100) | 99.5  (98.8-99.9) | 99.3  (93.2-99) | 100  (.-.) | 212  (79-164) | 0  (.-.) | 1.00 | 28.2 |

CI, confidence interval; LR+, positive likelihood ratio; LR-, negative likelihood ratio, NPV, negative predictive value; PPV, positive predictive value
