# Supplementary material for: Performance of mid-upper arm circumference as a screening tool for identifying adolescents with overweight and obesity
Source: PLoS One. 2020 Jun 23;15(6):e0235063. doi: 10.1371/journal.pone.0235063 (PMC7310830; doi:10.1371/journal.pone.0235063)
Supplement: S5 Table — (DOCX) [file pone.0235063.s007.docx]

Table 5. Ability of MUAC to classify obesity among adolescent, Addis Ababa,2019

| **Gender** | **AUC** | **SE** | **95% CI** |
| --- | --- | --- | --- |
| Males (n=456) | 0.99 | 0.0 | (0.98 - 1.00) |
| Females (n=395) | 0.97 | 0.01 | (0.95 – 0.99) |
| Total (n=851) | 0.98 | 0.01 | (0.97- 0.99) |

AUC, Area Under Curve; SE, Standard error; CI, Confidence interval
