# Supplementary material for: Performance of mid-upper arm circumference as a screening tool for identifying adolescents with overweight and obesity
Source: PLoS One. 2020 Jun 23;15(6):e0235063. doi: 10.1371/journal.pone.0235063 (PMC7310830; doi:10.1371/journal.pone.0235063)
Supplement: S6 Table — (DOCX) [file pone.0235063.s008.docx]

Table 6. Sensitivity, speciﬁcity, positive predictive value, negative predictive value, positive likelihood ratio, negative likelihood ratio, Youden index, and optimal cut-off values of mid-upper-arm circumference in predicting obesity (including obesity) (n=851)

| **Gender** | **Sensitivity (%)**  (95% CI) | **Specificity (%)**  (95% CI) | **PPV**  **(%)**  (95% CI) | **NPV**  **(%)**  (95% CI) | **LR+**  (95% CI) | **LR−**  (95% CI) | **Correctly**  **classified (%)** | **Youden index** | **Cut off point (cm)** |
| --- | --- | --- | --- | --- | --- | --- | --- | --- | --- |
| Males (n=456) | 100  (69.2-100) | 97.5  (95.6-98.8) | 47.6  (33.6-61.9) | 100  (.-.) | 40.5  (22.6-72.7) | 0  (.-.) | 97.37 | 0.98 | 31.9 |
| Females (n=395) | 100  (81.5-100) | 85.9  (82-89.3) | 25.3  (20.9-30.3) | 100  (.-.) | 7.11  (5.54-9.13) | 0  (.-.) | 86.58 | 0.86 | 28.6 |
| Total (n=851) | 96.4  (81.7-99.9) | 90.6  (88.4-92.5) | 26  (21.9-30.6) | 99.9  (99.1-100) | 10.3  (8.24-12.9) | 0.39  (0.01-0.27) | 90.25 | 0.87 | 29.5 |

CI, confidence interval; LR+, positive likelihood ratio; LR-, negative likelihood ratio, NPV, negative predictive value; PPV, positive predictive value
