# Supplementary material for: Performance of mid-upper arm circumference as a screening tool for identifying adolescents with overweight and obesity
Source: PLoS One. 2020 Jun 23;15(6):e0235063. doi: 10.1371/journal.pone.0235063 (PMC7310830; doi:10.1371/journal.pone.0235063)
Supplement: Attachment — Submitted filename: PLOS260220 Review of Performance of MUAC as a screening tool for identifying adolescents with overweight and obesity.doc [file pone.0235063.s009.doc]

**Review of Performance of MUAC as a screening tool for identifying adolescents with overweight and obesity**

I will now respond broadly and may be secondly more in detail. Starting with the selection of the participants. In fact there is nothing wrong with the procedure followed but the low number of participants makes it less suitable to draw conclusions. For instance it is not possible to calculate sex and age specific cutoff points, which is needed in the age period 15-19 years because of differences in (mean) MUAC per age category. I assume that the number of participants per age category, and sex, are insufficient to draw conclusions. Furthermore, representativeness is a major aspect, crucial, including SES (educational level of parents) for possible correction at national or urban level, this also applies to the student's educational level (low, middle, high). The training of the measurers is sufficiently.

The procedure to obtain the correct MUAC value is correct, similar that of BMI. I am having trouble choosing the WHO BMI Z score. For international comparison I prefer the BMI IOTF SDS (data from before the obesity epidemic. It is a pity that no distinction can be made between overweight (incl obesity; ow+) and obese children. (corresponding with age and sex specific BMI ≥ 25 and ≥ 30 kg/m² respectively). (low number of participants) The differences between overweight (excl obesity; ow-) and obesity could be of importance for guidance between the two groups. Comparison of MUAC with BMI is the only way there is, despite the flaws of BMI, but MUAC should primarily be compared with percentage body fat (%BF), although that is also not ideal (in vivo fat mass is not measurable accurately(Wells), and %BF is a ratio so assumptions have to be made (Goran/Allison))

The last overall aspect I will emphasize is ethnicity. For MUAC it is still not clear, but plausible that there are ethnically differences. I have no idea whether this is the case in AA (racial identical population??)

At first, this needs further explication, clarification, in the manuscript before drawing (jumping to) conclusions.

Secondly, I want more in detail comment the results, as the only-BMI (normal weight MUAC)and the only-MUAC (normal weight BMI), and the low agreement between BMI and MUAC in the age-group 2 - 5 years, because BMI is a weak measurement in this age-period (may be MUAC is even better)

However, this is an important study in a population other than Caucasian, where screening in a different way is much more difficult than in the western world.

Henk Talma, febr 20 2020
