# Supplementary material for: Performance of mid-upper arm circumference as a screening tool for identifying adolescents with overweight and obesity
Source: PLoS One. 2020 Jun 23;15(6):e0235063. doi: 10.1371/journal.pone.0235063 (PMC7310830; doi:10.1371/journal.pone.0235063)
Supplement: Attachment — Submitted filename: Response to Reviewers.docx [file pone.0235063.s010.docx]

**Referee(s)' Comments to Author:**

We would like to express our appreciation for reviewing the manuscript and your critical comments. We affirm that the manuscript has improved a lot from your comments.

**Point-by-point response_Reveiwer_01**

|  | Comments | Revision and response | Status |
| --- | --- | --- | --- |
|  | Abstract:   1. The acronym MUAC was used in line 22, then the authors should use it from this first mention (for example in lines 24, 26, 71, ...) 2. Line 38. Please add 95% CI for AUAC | 1. The Abstract section is revised based on your comments. 2. We have added 95% CI for AUC | Accommodated |
|  | Methods:   1. Line 138. It is necessary to offer more details about: If normality test was performed before the Spearman’s rank correlation? | 1. We checked the normality using formal test and graphically. We added more description about the normality test and its result in Line 150 – 153 of the revised manuscript with track change. | Accommodated |
|  | Results:   1. Line 184. Please add the meaning of the abbreviations BMI and MUAC in the footnote (Table 1). | 1. We have added the meaning of the abbreviations BMI and MUAC in the footnote. | Accommodated |
|  | Discussion:   1. Line 245-251. It is important to discuss the findings by comparing those with studies that evaluated adolescents aged over 15 years. For example: Mazıcıoğlu MM, Hatipoğlu N, Oztürk A, Ciçek B, Ustünbaş HB, Kurtoğlu S. Waist circumference and mid-upper arm circumference in the evaluation of obesity in children aged between 6 and 17 years. J Clin Res Pediatr Endocrinol. 2010; 2(4):144–150. doi:10.4274/jcrpe.v2i4.144 2. Line 274 – The limitations of the present study could be minimized by presenting the results of previous studies. For example, Taylor at al. concluded “Categorisation of BMI according to both age and pubertal stage of development does not produce cutoffs that are superior to BMI cutoffs calculated on the basis of age alone at identifying children with high DXA-measured adiposity”. | 1. We have discussed the findings of the study (Mazıcıoğlu MM, Hatipoğlu N, Oztürk A, Ciçek B, Ustünbaş HB, Kurtoğlu S. Waist circumference and mid-upper arm circumference in the evaluation of obesity in children aged between 6 and 17 years. J Clin Res Pediatr Endocrinol. 2010; 2(4):144–150. doi:10.4274/jcrpe.v2i4.144) in line number 248-249 2. We have revised the limitation according to the findings of (Taylor et al., 2003). | Accommodated |

**Point-by-point response_Reveiwer_02**

|  | Comments | Revision and response | Status |
| --- | --- | --- | --- |
|  | 1. The last overall aspect I will emphasize is ethnicity. For MUAC it is still not clear, but plausible that there are ethnically differences. I have no idea whether this is the case in AA (racial identical population??) | 1. Addis Ababa's population has a somehow homogeneous racial identity. In fact, ethnicity could have an impact on anthropometric measurements (both MUAC and BMI). However, we consider it as random variation. As our study focused narrow range of age groups, the effect is expected to be minimal. Ethnicity is not biological (body fat and fat free mass distribution) rather it is cultural. For this reason, we don’t believe ethnicity specific cut off points will provide any addition benefit. | Accommodated |

**Point-by-point response_Reveiwer_3**

|  | Comments | Revision and response | Status |
| --- | --- | --- | --- |
|  | 1. I have serious reservations with the premise on which it is based which is that the BMI-for-age cut-off for overweight and obesity is an appropriate definition against which to calibrate an equivalent MUAC cut-off. | 1. We also share the concern of the reviewer about using BMI Z score as a reference. Even though BMI Z score has its own limitation, it is correlated with percent body fat (Garrow and Webster, 1985, Freedman et al., 2013, Wohlfahrt-Veje et al., 2014)BMI Z score is the indicator which is accepted WHO to quantify the burden of obesity and overweight in resource areas where there are limited alternatives to estimate prevalence of obesity and overweight (de Onis et al., 2007). In Ethiopia BMI z score of the only indicators which has been used to determine the magnitude of obesity and overweight among adolescents. Also, it’s the most commonly used tool and the only available method in resource-limited settings like Ethiopia. For this reason, we decide to use BMI Z score as a reference test. | Accommodated |
|  | Abstract   1. line 38 characteristic singular | 1. Thank you. This has been corrected | Accommodated |
|  | Introduction   1. line 47 change 'calorie' to energy (calorie is the unit of measurement not the variable) 2. line 67 adolescent (no s) | 1. The comment is accepted and we have modified them accordingly. 2. Thank you. This has been corrected | Accommodated |
|  | Methods   1. It would be important to explain/justify the rationale for calibrating cut-offs against BMI for age defined overweight and obesity in general. 2. why calibrate against overweight as opposed to obesity (since obesity is more important, and has been related to comorbidities in a way which overweight has not). 3. It would have been useful to follow explicitly the guidance on studies of this kind which are available e.g. STARD. 4. Line 127 delete 'Adolescents with'. 5. It would be useful to define/explain concepts and terms such as 'optimism' here. The other concepts and terms are much better known but it would also be useful to define these briefly in the text: sensitivity; specificity; positive and negative predictive values. | 1. We added a couple of lines explain the rationale for calibrating cut-offs against BMI for age defined overweight and obesity in general (line 132-136) in revised manuscript with track change. 2. This has been included in the limitation of the study 3. The manuscript has been revised according to STARD 2015 guideline. We have attached STARD 2015 checklist as supplementary file. 4. Thank you so much for the careful eyes. This has been corrected accordingly. 5. We have defined in briefly: sensitivity; specificity; positive and negative predictive values.   We also add a description how computed the optimism coefficient (line 166,168) of the revised manuscript with track change. | Accommodated |
|  | Results   1. Line 197 should be characteristic singular 2. Line 198 should be was not is (past tense). 3. Lines 205-207 notes excellent agreement but clarify that this is at the optimum cut-off point. | 1. Thank you so much for the careful eyes. This has been corrected accordingly. 2. Thank you so much for the careful eyes. This has been corrected accordingly. 3. We think that the reviewer misunderstood it. The ROC curve and associated AUC do not use specific cutoff point. The ROC curve is plotted the true positive rate (TPR) against the false positive rate (FPR) at various threshold or cutoff. The optimal cutoff is determined using Youden index. Then, other performance measures such as specificity, sensitivity, PPV, NPV, LRs, are calculated using the optimal cutoff point. | Accommodated |
|  | Discussion   1. While the authors acknowledge the weakness of not calibrating MUAC against a reference method (gold standard) on lines 269-272 I don't think they go nearly far enough here and the implications of their findings are potentially harmful. Since BMI-for age performs poorly as a proxy for obesity (obesity is excessive fatness and BMI for age has only low-moderate sensitivity but high specificity according to multiple systematic reviews) calibrating a MUAC cut point which is equivalent to a BMI for age cut point would simply replace one poor proxy by another. For that reason, I think that this is a fundamental conceptual flaw in this study event though it is otherwise sound methodologically. 2. I also note that alternative methods of measuring body fatness more directly (DEXA is mentioned in the Discussion) are not reference methods, e.g. see Wells and Fewtrell Arch Dis Child 2006. The only reference or gold standard lab methods are multi-component and the only field reference method is total body water. 3. Line 245 adolescent singular. | 1. We appreciate the concern of the reviewer. It is true that replacing BMI for age with MUAC would have a similar limitation in terms of identifying obesity and overweight adolescents. But still, BMI Z score is the widely used tool if not the only tool in nutrition surveys in resource-limited settings, so we want to evaluate if the MUAC has equivalence accuracy with BMI Z score so that it can be used for screening in large scale surveys. MUAC has some advantages over BMI Z score in terms of practicality. That is why we appreciate the limitation of our approach and emphasized the need for further study to evaluate the performance of MUAC in relative to gold standard methods. 2. Thank you so much for the careful eyes. We have revised the limitation part accordingly. 3. Thank you so much for the careful eyes. This has been corrected accordingly. | Accommodated |

**References**

DE ONIS, M., ONYANGO, A. W., BORGHI, E., SIYAM, A., NISHIDA, C. & SIEKMANN, J. 2007. Development of a WHO growth reference for school-aged children and adolescents. *Bull World Health Organ,* 85**,** 660-667.

FREEDMAN, D. S., HORLICK, M. & BERENSON, G. S. 2013. A comparison of the Slaughter skinfold-thickness equations and BMI in predicting body fatness and cardiovascular disease risk factor levels in children. *The American journal of clinical nutrition,* 98**,** 1417-1424.

GARROW, J. S. & WEBSTER, J. 1985. Quetelet's index (W/H2) as a measure of fatness. *International journal of obesity,* 9**,** 147-153.

TAYLOR, R. W., FALORNI, A., JONES, I. E. & GOULDING, A. 2003. Identifying adolescents with high percentage body fat: a comparison of BMI cutoffs using age and stage of pubertal development compared with BMI cutoffs using age alone. *Eur J Clin Nutr,* 57**,** 764-9.

WOHLFAHRT-VEJE, C., TINGGAARD, J., WINTHER, K., MOURITSEN, A., HAGEN, C. P., MIERITZ, M. G., DE RENZY-MARTIN, K. T., BOAS, M., PETERSEN, J. H. & MAIN, K. M. 2014. Body fat throughout childhood in 2647 healthy Danish children: agreement of BMI, waist circumference, skinfolds with dual X-ray absorptiometry. *Eur J Clin Nutr,* 68**,** 664-70.
