# Supplementary material for: Performance of mid-upper arm circumference as a screening tool for identifying adolescents with overweight and obesity
Source: PLoS One. 2020 Jun 23;15(6):e0235063. doi: 10.1371/journal.pone.0235063 (PMC7310830; doi:10.1371/journal.pone.0235063)
Supplement: Attachment — Submitted filename: tweede review MUAC Ethiopie I refer firstly to my letter from febr 20.doc [file pone.0235063.s011.doc]

I refer firstly to my letter from febr 20, 2020 about the low number of participants, for age specific representative data you require at least about 200 participants per age category and sex because there are still significant (growth) changes in height and MUAC in adolescence (15-19 years). Mean age shows that there are more youngsters than 18 and 19 year olds in this study.

In terms of representativeness there is still no clarity regarding the educational level of the participants, high school means one educational level (high?), no middle or low level education? In case it will be necessary to correct for the national situation. This is also in case you have to correct for the national situation for the educational level of the parents (or the city of AA).

The homogeneous racial identity excludes possible ethnic differences.

Comparing MUAC with BMI is scientifically a weakness, but mostly the only way to perform a study like this, then BMI IOTF is the best to compare with. For determining %BF (foot-to-foot) BIA is in the Ethiopian setting possibly a suggestion.

This study should be repeated in several years (5-10), using the exact same/identical (measuring) protocol with a larger population, to establish trends especially in **obese** adolescents. We discovered in comparing WC with BMI a threefold higher % obesity with BMI than with WC, meaning? (BMI distinguishes obesity less well?) So it is not said when MUAC predicts obesity equally as BMI, that MUAC is equal to BMI, MUAC could be even better or worse in predicting obesity. May be comparing MUAC with %BF could reveal this. BMI is tricky to compare with. MUAC is an expression of total and general fat mass in another way than BMI, as is WC, therefore it is indeed sensible to evaluate the validity of MUAC to identify adolescents with health risks. In case of Child Public Health early detection of **obesity** is most valuable (less large numbers, less false positives, less false negatives (numbers)) for treatment and guidance in connection with health risks.

The most interesting is table 2 with the only-BMI (normal weight MUAC and overweight BMI) and the only-MUAC (normal weight BMI and overweight MUAC). The total agreement between BMI and MUAC is 90.4%.The only-BMI is 1.3%, the false positives (?), partly the MHO (metabolically healthy obese?). The only-MUAC is 8.3%, the false negatives(?), partly the MONW (metabolically obese normal weight?), the latter group is the most interesting in child public health (are at risk but not detected). As long as there is no real comparison is possible between BMI and MUAC, a study where MUAC and BMI are independently are compared with a gold standard for %BF (in the same study) it will be unclear if MUAC may discriminate in obesity ( instead of overweight incl obesity, preferably obesity) better than BMI or even worse. This gold standard could be (foot-to-foot BIA if the used device is calibrated with DEXA, so corrections can be made.

WHO 2007 Growth charts, comparison BMI WHO and BMI IOTF cutoffs

**Interpretation of cut-offs WHO**

Overweight: > +1 SD (equivalent to BMI 25 kg/m^2^ at 19 years)
Obesity: > +2 SD (equivalent to BMI 30 kg/m^2^ at 19 years)
Thinness: < -2 SD >
Severe thinness: <>

This comparison of growth charts shows the differences between the two reference charts, revealing/meaning they are not just comparable (BMI WHO, and BMI IOTF), group overweight incl obesity (ow+) is larger in WHO as in IOTF, although in the age group 15-19 y it is more comparable than in the 5-14 y (boys and girls; that is logic because they match the adult BMI respectively at 19 and 18 years ), in the group obesity the differences are even larger, this may be due to the used data (WHO) being partly gathered during the obesity epidemic (are not from before 1980). So, the one BMI is not the other, this are different sorts of apples (instead of oranges). As said, BMI in different studies gives different results, and BMI scores higher percent obesity of which about 50% is false positive (Romero-Corral 2007 in adults), so other anthropometric measurements (instruments) that can discriminate better than BMI, like possibly MUAC, we urgently need. So comparing with BMI does not help us forward (beyond BMI). See ref 28 Mazicioglu: ‘Despite the scientific flaws of BMI, as there is no general consensus on reliability, use, and application of direct and indirect anthropometric indices describing obesity in children, BMI will remain essential for diagnosing obesity in the future, but additional anthropometric indices are needed to describe body fat distribution more accurately’. Mazicioglu 2010

It is a sober study, but worth publishing, because it presents the limitations of (field) research in developing countries, data we needlessly lack. It illustrates that scientific choices not can be made so comparisons are unbalanced. We have to provide them with simple instruments (like foot-foot BIA for determining %BF) to perform field research, making it possible to compare the correct parameters. In research it is of outmost importance using, for instance, the same/identical protocol, the representativeness, enough statistical power, and so on. We need more insight in the real situation in those countries. This study would gain strength when there was a choice not for one cutoff point for the whole age range, but age specific from 15 to 19 years. And not only for overweight incl obesity (ow+; overweight +), but more specific for obesity (> 2 SD). And using BMI IOTF instead of BMI WHO, for international comparison.

Henk Talma

March 09, 2020
