# Supplementary material for: Performance of mid-upper arm circumference as a screening tool for identifying adolescents with overweight and obesity
Source: PLoS One. 2020 Jun 23;15(6):e0235063. doi: 10.1371/journal.pone.0235063 (PMC7310830; doi:10.1371/journal.pone.0235063)
Supplement: Attachment — Submitted filename: Response to Reviewers.docx [file pone.0235063.s012.docx]

**Referee(s)' Comments to Author:**

We would like to express our appreciation for reviewing the manuscript and your critical comments. We affirm that the manuscript has improved a lot from your comments.

**Point-by-point response_Reveiwer_02**

|  | Comments | Revision and response | Status |
| --- | --- | --- | --- |
| 1 | In terms of representativeness there is still no clarity regarding the educational level of the participants, high school means one educational level (high?), no middle or low level education? In case it will be necessary to correct for the national situation. This is also in case you have to correct for the national situation for the educational level of the parents (or the city of AA). | A high school in Ethiopia is equivalent to secondary level education, and encompasses “grade 9 to 12”. |  |
| 2 | This study would gain strength when there was a choice not for one cutoff point for the whole age range, but age specific from 15 to 19 years. | Your comment is absolutely right and we understand your concern. Age and sex-specific cut off points would indeed have much better performance. However, due to the small sample size in the present study, the age and sex-specific cutoff points are not precise. We have acknowledged this limitation in the paper (line number 293-296). For readers, we have included the age and sex specific cutoffs in the supporting information as supplemental material. |  |
| 3 | And not only for overweight incl obesity (ow+; overweight +), but more specific for obesity (> 2 SD). | We also agree the paper would gain more strength if we also included cutoff points for obesity. This is true as obesity more related to metabolic syndrome and adverse health outcomes. However, since there were small number of obese adolescents included in this study, it was not possible to determine cutoff point for obesity. We have acknowledged this limitation in the current version of the manuscript. |  |
| 3 | And using BMI IOTF instead of BMI WHO, for international comparison. | Dear reviewer, we had considered IOTF as a reference standard. However, we were not able to use the IOTF because it is applicable for only 2-18 years old (Cole et al., 2000). However, in this study we included adolescents aged 19 years old which can't be compared using IOTF. For this reason, we choose the WHO 2007 BMI Z score as a reference standard. |  |

**Point-by-point response_Reveiwer_03**

|  | Comments | Revision and response | Status |
| --- | --- | --- | --- |
| 1 | The authors have partially addressed the major concern that I had at the previous review stage, that use of BMI Z score as the reference method was limited because that is a poor indicator of excess fatness. The authors now acknowledge this important point in the revised discussion, but the language used in places in the Discussion still tends to ignore this difficulty and in doing so overstates the accuracy of MUAC. In particular Discussion lines 252 and 255 overstate accuracy- accuracy of MUAC cannot be 'excellent' because it is being judged against a reference method (BMI Z score) which itself is not excellent. In summary, the authors should use more careful, appropriate, language in lines 252 and 256, or should omit these exaggerated claims. | We have revised the discussion section as recommended (line number 253-254). | Accommodated |
| 2 | Line 288- the gold standard is not 'doubly labelled water' and so this phrase should be replaced with ' total body water or multi-component methods (which measure body density, total body water, and total body mineral'. | This is accommodated. | Accommodated |
| 3 | The manuscript is generally well written and clear, but there are a few minor grammatical errors and I think it would benefit from an English language edit. | We have revised the whole manuscript for spelling and grammar. | Accommodated |

COLE, T. J., BELLIZZI, M. C., FLEGAL, K. M. & DIETZ, W. H. 2000. Establishing a standard definition for child overweight and obesity worldwide: international survey. *BMJ,* 320**,** 1240-3.
